# Supplementary material for: Plant Poly(ADP-Ribose) Polymerase 1 Is a Potential Mediator of Cross-Talk between the Cajal Body Protein Coilin and Salicylic Acid-Mediated Antiviral Defence
Source: Viruses. 2023 May 30;15(6):1282. doi: 10.3390/v15061282 (PMC10300765; doi:10.3390/v15061282)
Supplement: Supplementary file 1 [file viruses-15-01282-s001.zip › Table S1.pdf]

Table S1. Primers used for quantitative RT-PCR

Primer	5'-3' sequence	Reference	Primer concentration (nM)	E (%)
NbUBI3-F<br>NbUBI3-R	AATGTGAAAGCCAAGATCCAAG<br>CGGAGGCGGAGCACGAGATGAA	GenBank<br>TC20187	300	91.2
NbL23-F<br>NbL23-R	AAGGATGCCGTGAAGAAGATGT<br>GCATCGTAGTCAGGAGTCAACC	GenBank<br>TC19271	350	97
TRV RNA1-F<br>TRV RNA1-R	CAGTCTATACACAGAAACAGA<br>GACGTGTGTACTCAAGGGTT	GenBank<br>AF406990.1	350	94
NbPR-1a-F<br>NbPR-1a-R	CCGTTGAGATGTGGGTCAAT<br>CGCCAAACCACCTGAGTATAG	GenBank<br>JN247448.1	350	98
NbPARP1-F<br>Nb PARP1-R	AACTGGAGGCACAGACTAAAGCCT<br>ACTGAGATAGCCCATTAGCAGCCTG	GenBank<br>KP771975	340	97
