# Supplementary figures and images for: Plant Poly(ADP-Ribose) Polymerase 1 Is a Potential Mediator of Cross-Talk between the Cajal Body Protein Coilin and Salicylic Acid-Mediated Antiviral Defence
Source: Viruses. 2023 May 30;15(6):1282. doi: 10.3390/v15061282 (PMC10300765; doi:10.3390/v15061282)

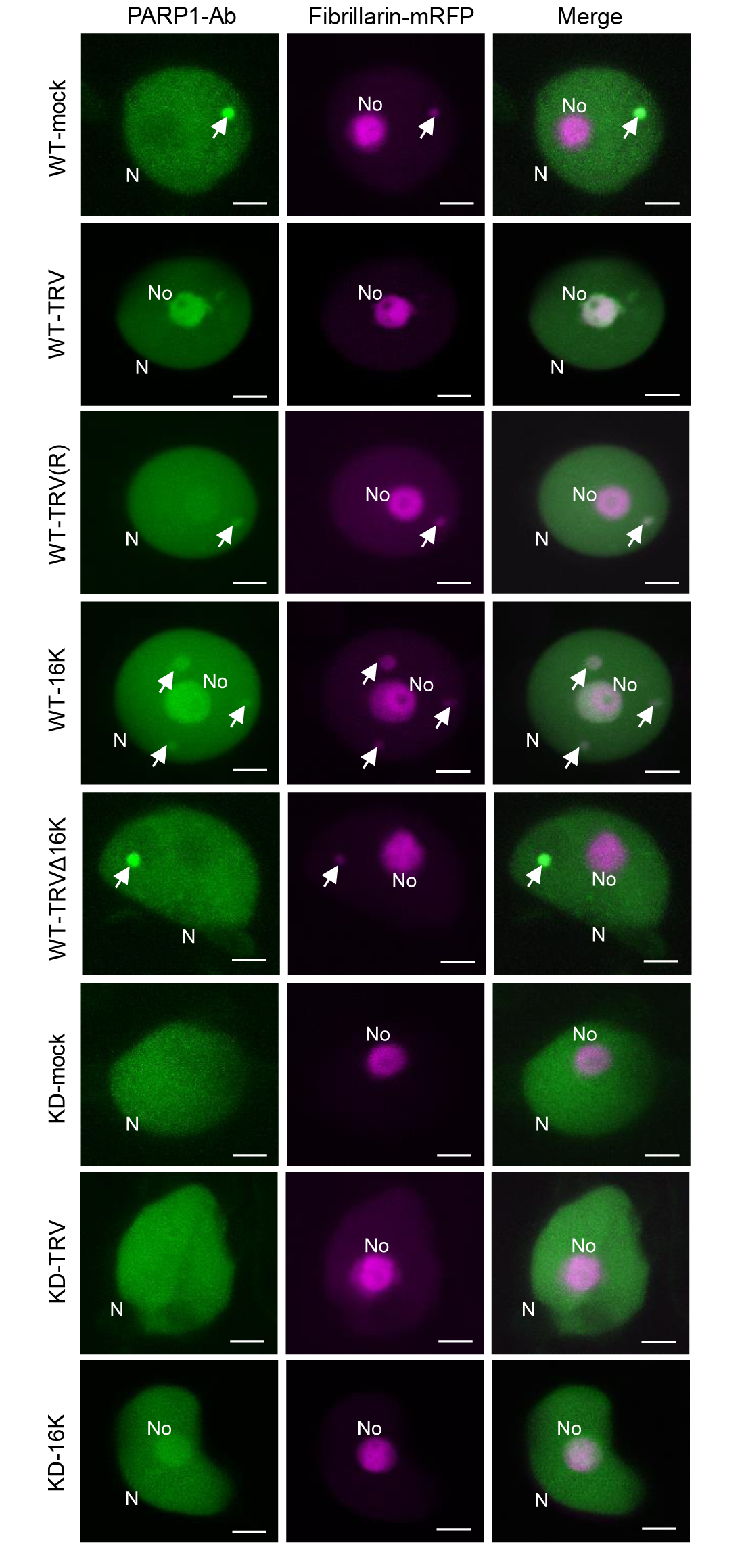

Supplement: Supplementary file 1 [file viruses-15-01282-s001.zip › Figure S1.tif]
